# Immunization Agenda 2030 and Brazil’s challenges

**DOI:** 10.1590/S2237-962220230003000018.EN

**Published:** 2023-12-04

**Authors:** Cynthia Braga, Barbara Reis-Santos

**Affiliations:** 1 Fundação Oswaldo Cruz, Instituto Aggeu Magalhães, Recife, PE, Brazil; 2 Rede Brasileira de Pesquisas em Tuberculose, Rio de Janeiro, RJ, Brazil

The decline in vaccine coverage and the subsequent resurgence of vaccine-preventable diseases have posed a significant global challenge.^1^ Lack of access to vaccines and vaccine hesitancy - defined as reluctance or refusal to vaccinate despite the availability of vaccines - have contributed to a decrease in vaccine coverage worldwide over the past decade.^2^ This downward trend has been worsened due to the dramatic consequences of the COVID-19 pandemic, which had a counterproductive impact on efforts to increase vaccine coverage in most countries.^3^

In 2022, despite the intensification of immunization actions and the end of the COVID-19 pandemic, global coverage of the yellow fever vaccine was 48% in endemic countries, and the first dose of HPV vaccine among girls was only 21%, well below the 80% coverage that is recommended.^3^ During the same period, Brazil ranked among the top ten countries in the world, most of them located in Africa and Asia, where approximately 60% of the 20 million children had not received the first dose of DTP (diphtheria, tetanus and pertussis) vaccine or were partially vaccinated.^2^

In response to this severe global health threat, the World Health Organization (WHO), in collaboration with other partner institutions, proposed the Immunization Agenda 2030 (IA2030). Launched in 2020, this strategy aims to maximize the impact of vaccines on a global scale by strengthening national immunization programs.^4^ Among the Agenda’s key goals is achieving 90% vaccination coverage for essential vaccines administered in childhood and adolescence, and halving the number of children completely missing out on vaccines, by 2030.^4^ ^),(^ ^5^

In order to achieve these goals, prioritization, improvement of strategies and operational planning, and vaccination data monitoring at the local level are of utmost importance, aiming to ensure access to vaccine products and the prevention of vaccine-preventable diseases among populations.^5^

The global trend of the decrease in vaccination coverage, in recent years, has been intensified in Brazil during the COVID-19 pandemic, to the extent that it poses a threat to the results and success of the National Immunization Program (*Programa Nacional de Imunizações* - PNI) over its nearly five decades of existence. The analyses conducted by Moura et al. - using data from the National Immunization Program Information System (*Sistema de Informações do Programa Nacional de Imunizações* - SI-PNI) and the Live Birth Information System (*Sistema de Informações sobre Nascidos Vivos* - SINASC), presented in an article published in this issue of Epidemiology and Health Services (*Epidemiologia e Serviços de Saúde* - RESS) journal,^6^ are clear evidence of downward trend in vaccination coverage and increasing dropout rates of the triple viral vaccine in Brazil, from 2014 to 2021. This evidence points to the significant challenges that the country will face in the coming years in achieving IA 2030 goals.

It is worth highlighting the potential of the PNI in addressing this problem. This Program, internationally recognized, celebrates 50 years of existence, marked by accumulated experience, progress and success in projected goals, especially in overcoming challenges. Beyond its virtuous aspects, the prospects for the PNI in the coming years were discussed in an editorial note in RESS.^7^ The Program has a physical and technical-administrative structure of the cold chain network integrated with the Primary Health Care network within the Brazilian National Health System (*Sistema Único de Saúde* - SUS), at various levels of management, across the entire national territory.^8^ It is worth noting, despite limitations related to data timeliness and quality,^9^ the usefulness, flexibility and coverage of the National Immunization Program Surveillance System, which enables continuous data collection and monitoring of vaccination coverage and dropout across the country, as an important tool for planning PNI actions at the local and regional scales within different levels of SUS management. Notwithstanding the national autonomy in the vaccine production and its contribution to the population access to vaccines, significant technological challenges and production bottlenecks persist, aimed at reducing and eliminating our dependence on countries that traditionally produce these inputs.^10^

In addition to the current scenario of increasing vaccine hesitancy and consequent decline in coverage, there is the spread of fake news.^11^ ^),(^ ^13^ Ensuring universal access to vaccines for the population requires complex and diverse actions, such as planning and investment in skills training, strengthening infrastructure, and scientific and technological development at the various organizational levels. In order for these actions to be successful and for the National Immunization Program to regain trust and adherence of the population, built over the years, the commitment of the scientific community, health professionals, non-governmental organizations and the government is essential. Only with the involvement of organized society will it be possible to guide the country back to its position as a global leader in vaccination actions.
